# Motivation of pediatricians in Lower Saxony to teach medical students in outpatient practices: A questionnaire-based study

**DOI:** 10.3205/zma001760

**Published:** 2025-06-16

**Authors:** Alexandros Rahn, Thomas Müller, Benjamin Stadlbauer, Anna-Lena Herbach, Lennart Greiner, Urs Mücke

**Affiliations:** 1 Medizinische Hochschule Hannover, Hannover, Germany

**Keywords:** pediatrics, teaching motivation, teaching practice, network, change management

## Abstract

**Objective:**

This study examines the teaching motivation of pediatricians working in outpatient settings in Lower Saxony, aiming to promote medical student training in outpatient teaching practices. The focus lies on identifying motivating factors and barriers in order to develop concepts for establishing a “pediatric teaching network”.

**Methods:**

A digital, anonymous questionnaire survey was conducted between July and September 2024. The cross-sectional study, based on prior work from general practice, included 27 items addressing intrinsic and extrinsic motivation, as well as barriers related to student teaching. Responses were rated on a four-point Likert scale and supplemented with open-ended questions. Univariate analysis methods were applied, and factors influencing teaching motivation were explored.

**Results:**

A total of 137 complete responses were evaluated. Given the total number of potential respondents, the generalizability of the results may be limited. The average teaching motivation was 7.5 out of 10 points. Intrinsic motives – such as promoting the next generation (99% agreement) and knowledge exchange (98% agreement) – were dominant. Extrinsic incentives, such as recognition as an “academic teaching practice” (78% agreement), were considered relevant, while financial incentives were of lesser importance. The main barrier identified was concern that students might disrupt practice operations (53% agreement).

**Conclusion:**

The strong intrinsic teaching motivation of pediatricians indicates potential for developing pediatric teaching networks. However, targeted collaboration is needed to overcome structural barriers, such as integrating students into routine practice. Strategies to enhance teaching motivation include (medical) didactic training, flexible teaching models, and organizational support.

## Zusammenfassung

**Zielsetzung:**

Die Studie untersucht die Lehrmotivation ambulant tätiger Kinderärzt:innen in Niedersachsen mit dem Ziel, die Ausbildung von Medizinstudierenden in Lehrpraxen zu fördern. Im Fokus stehen die Identifikation von Motivationsfaktoren sowie Barrieren, um Konzepte zur Gründung eines „Netzwerk Lehre Pädiatrie“ zu entwickeln.

**Methodik:**

Eine digitale, anonyme Fragebogenerhebung wurde von Juli bis September 2024 durchgeführt. Die auf Vorarbeiten aus der Allgemeinmedizin basierende Querschnittsstudie umfasste neben Freitexten 27 Items zu Motivation, Anreizen und Hürden der studentischen Lehre, die mittels einer vierstufigen Likert-Skala bewertet wurden. Es wurden univariate Analyseverfahren verwendet. Einflüsse auf die Lehrmotivation wurden untersucht.

**Ergebnisse:**

137 vollständige Rückmeldungen wurden ausgewertet, was in Hinblick auf die Gesamtzahl möglicher Antworten eine begrenzte Aussagekraft bedingen kann. Die Lehrmotivation lag bei durchschnittlich 7,5 von 10 Punkten. Intrinsische Motive, wie die Nachwuchsförderung (99% Zustimmung) und der Wissensaustausch (98% Zustimmung), dominierten. Extrinsische Anreize, wie die Aufwertung als „Akademische Lehrpraxis“ (78% Zustimmung), wurden als relevant erachtet, während finanzielle Anreize weniger Bedeutung hatten. Hauptbarriere waren Befürchtungen, dass Studierende den Praxisablauf stören könnten (53% Zustimmung).

**Schlussfolgerung:**

Die intrinsische Lehrmotivation von Kinderärzt:innen zeigt Potenzial für den Aufbau pädiatrischer Lehrnetzwerke, erfordert jedoch gezielte Zusammenarbeit zur Überwindung struktureller Barrieren wie der Integration Studierender in den Praxisablauf. Ansätze zur Verbesserung der Lehrmotivation umfassen (medizin-) didaktische Fortbildungen, flexible Lehrmodelle und organisatorische Unterstützung.

## 1. Introduction

The “Master Plan for Medical Studies 2020” (MPMS 2020), adopted in 2017, includes a total of 37 measures aimed at reforming medical education in Germany. Its goal is to ensure that the training of future generations of physicians is adapted to challenges such as demographic change and the shortage of healthcare professionals in rural areas. A central theme of the reform is competence-based education, which focuses not only on the acquisition of knowledge but also on the development of clinical skills and professional attitudes [1]. One proposed solution is the increased integration of teaching practices into medical education, as is already common in general practice. As early as 2014, the GMA Primary Care Committee published a position paper on training in the primary care sector, which included comprehensive recommendations for pediatrics [2]. In its detailed statement on MPMS 2020, the Teaching Working Group of the German Society for Pediatrics and Adolescent Medicine (DGKJ) strongly advocates for the recruitment of pediatric teaching practices, emphasizing that outpatient medicine must be more prominently represented in the curriculum [3].

In addition, the authors’ experience suggests that students cannot adequately learn how to conduct pediatric check-ups for children of various ages (U1-J2) through hospital-based learning experiences alone. Early exposure to primary care is also essential for the development of students’ professional roles and identity [4]. A potential, though not yet widely implemented, solution could be the establishment of a network of pediatric teaching practices. While teaching in general practice settings has long been an integral part of medical education [5], according to the authors’ knowledge, currently only 14 out of 33 members of the German Medical Faculty Association (MFT) who responded to a survey – out of a total of 39 – have pediatric teaching practices or individual cooperations.

In recent years, the workload for office-based pediatricians has significantly increased due to various factors, such as a growing number of mandatory health check-ups and the rising prevalence of chronic diseases among children and adolescents [6]. Since student training usually takes place in addition to regular patient care, a high level of teaching motivation among physicians is a key prerequisite for implementing such a teaching project. While numerous national and international studies have demonstrated strong motivation for teaching in the field of general practice, there is a lack of comparable data for pediatrics [7], [8], [9]. Following the 2023 study by Daunert et al. [5], which surveyed over 500 general practitioners in Thuringia to establish an “evidence base for concepts and recommendations on recruiting teaching physicians”, a questionnaire was developed to assess both intrinsic and extrinsic motivation for undergraduate teaching among office-based pediatricians and outpatient pediatric trainees in Lower Saxony, as well as to identify barriers to student teaching [5].

Here, intrinsic motivation refers to motivation driven by internal incentives (e.g., interest, personal values), while extrinsic motivation is shaped by external rewards (e.g., recognition, praise) [10]. 

### 1.1. Objective

Competence-based medical education requires that students engage with patients not only in university settings but also in outpatient care. Therefore, Measure 15 of the MPMS 2020 explicitly calls for the recruitment of additional academic teaching practices [1]. However, it remains unclear what prerequisites already exist in pediatrics or what conditions need to be established.

The guiding questions for this survey were:

- What level of motivation for undergraduate teaching exists among office-based pediatricians and outpatient pediatric trainees?
- What influence do defined motivational factors or barriers have?
- What insights can be gained regarding participation in undergraduate teaching in pediatric primary care in Lower Saxony?

## 2. Methods

### 2.1. Questionnaire

The questionnaire was developed based on the work of Daunert et al. [5] and Adarkwah et al. [11]. Although detailed information on psychometric quality criteria was not available, pretests were conducted in both the preliminary studies and the present investigation to ensure quality. The questionnaire was made available online for a three-month period (07/2024-09/2024) using the platform “SoSci Survey” (SoSci Survey GmbH, Munich, Germany).

In addition to ten sociodemographic questions, a total of 27 items were collected anonymously across the domains of motivation, incentives, and barriers related to undergraduate teaching. Most of the questions were designed to be answered using a four-point Likert scale. The Ethics Committee of Hannover Medical School raised no objections to the study (May 30, 2024, No. 11430_BO_S2024).

### 2.2. Data analysis

An invitation to participate in the online survey was sent by post in July 2024 to 363 pediatric practices registered with the Association of Statutory Health Insurance Physicians of Lower Saxony (KVN) and remained accessible for a period of three months. Distribution also took place via an email mailing list and a featured editorial article in the “Niedersächsisches Ärzteblatt” (09/2024). No compensation was offered. Participation was voluntary. The returned questionnaires were exported to an Excel file (Microsoft^®^ Excel, Version 16.90, Washington, USA) and manually checked for completeness.

For statistical analysis, Likert scale responses (ordinal scaling) were dichotomized as follows: agreement (“strongly agree” and “somewhat agree”) and disagreement (“strongly disagree” and “somewhat disagree”) [12]. In the univariate analysis, the item “How motivated are you to train medical students in your practice?” (scale from 0 to 10) was defined as the target variable. Sociodemographic data (e.g., age, gender, duration of medical practice) as well as items on intrinsic and extrinsic motivation and perceived barriers were treated as influencing variables.

GraphPad Prism 8 (GraphPad Software, Boston, Massachusetts, USA) was used for statistical analysis. The Mann-Whitney U test for unpaired samples (U test) was used to determine asymptotic significance (p-value). Additionally, the overall sample was divided into “experienced” and “inexperienced” groups based on the item “I have already had experience with students during my time in outpatient practice,” and analyzed using Fisher’s exact test. In both cases, differences with a p-value<0.05 were considered statistically significant.

## 3. Results

Of the 151 returned questionnaires, 137 were included in the analysis, as 14 were incomplete and not finalized. On average, 12% of questions were left unanswered. When the four open-ended questions were excluded, the average rate of missing responses was only 2.5%. More than half of the participants were female (58%), and 46% of respondents were aged 56 years or older. A total of 44% had been practicing as outpatient physicians for 16-35 years. Practice owners accounted for 84% of respondents. A subspecialty certification was held by 42%. While examination rooms suitable for teaching were available in 8 out of 10 practices, only about half of the respondents (54%) had formal authorization to provide postgraduate training.

Overall, 80% reported having gained experience with medical students during their specialist training, and three-quarters of the physicians had supervised students during their time in outpatient practice (see table 1 (Tab. 1)).

### 3.1. Descriptive analysis

The average teaching motivation score was 7.5 points (range: 2 to 10) on a scale from 0 (no motivation) to 10 (high motivation). No significant differences in overall teaching motivation were found in relation to age or gender. Interest in training medical students was reported by 97%. Only 2% of respondents reported having had negative experiences with students. However, it cannot be excluded that response bias influenced these answers due to a potential desire to provide socially desirable responses.

**Figure 1:**
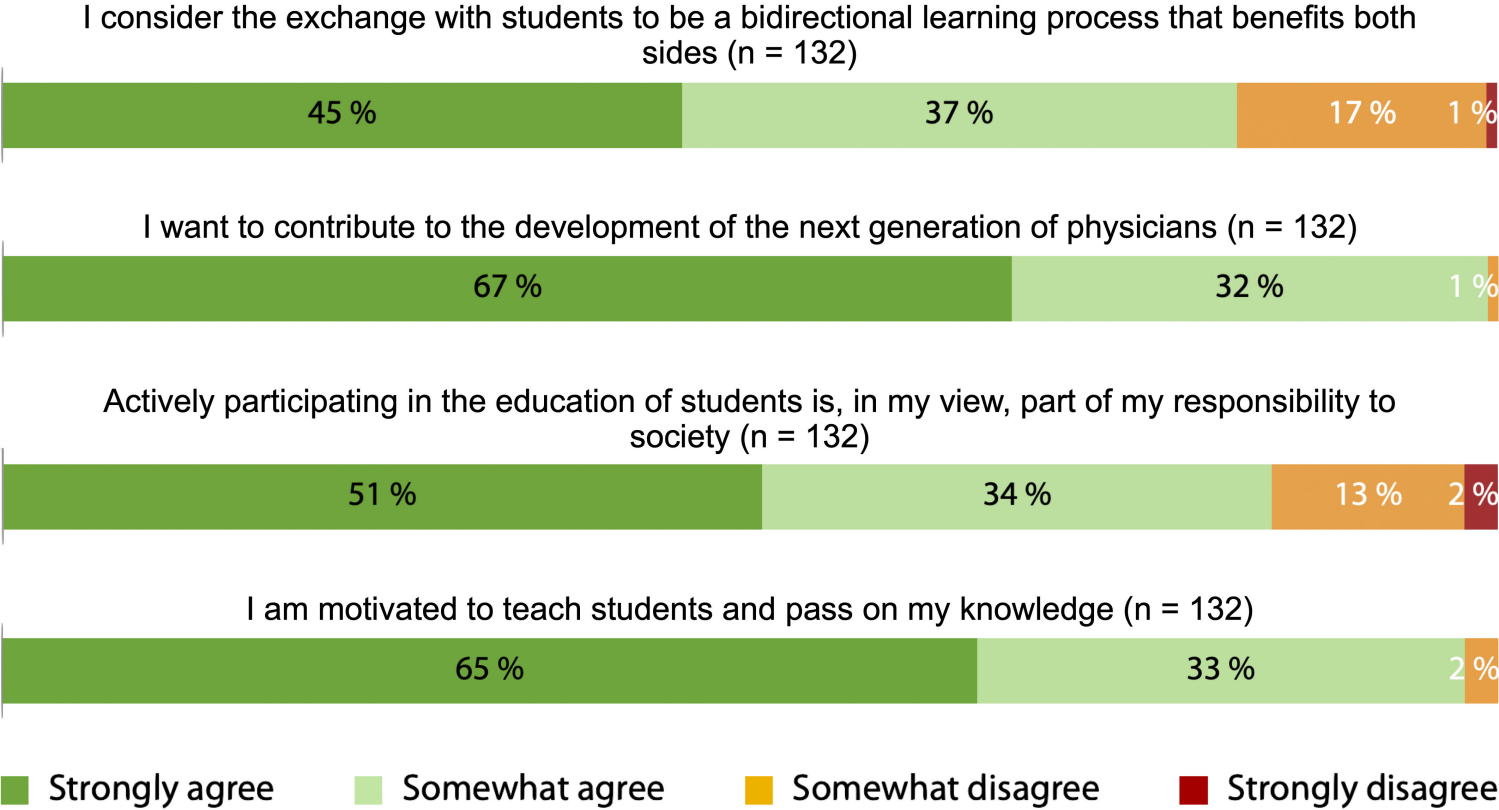
Agreement with intrinsic motives for participating in student teaching based on a 4-point Likert scale

**Figure 2:**
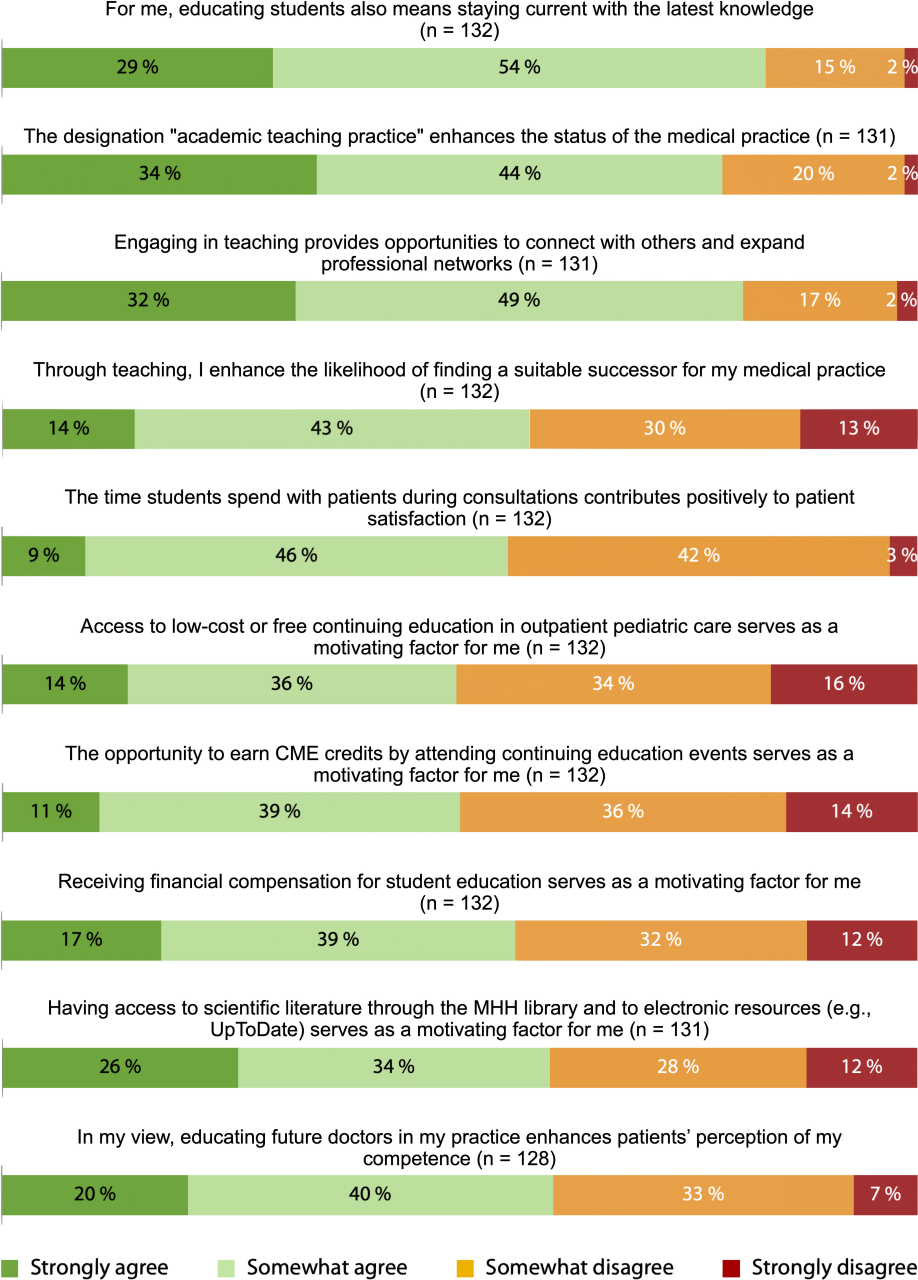
Agreement with extrinsic motives for participating in student teaching based on a 4-point Likert scale

The agreement with the four intrinsic motivation items was very high, with 99% agreeing that they contributed to fostering future talent, 98% expressing a desire to share knowledge, 85% feeling a sense of responsibility toward society, and 82% supporting knowledge exchange (see figure 1 (Fig. 1)). Regarding extrinsic motivation, the highest agreement was observed for staying up-to-date with the latest knowledge (84%), networking (81%), recognition through the designation of “academic teaching practice” (78%), and access to professional literature (60%). These factors represented the most frequent extrinsic incentives for participating in medical student teaching. Conversely, incentives such as financial compensation as a motivating factor (56%), participation in cheaper or free educational courses, and earning CME points (both 50%) received significantly less support (see figure 2 (Fig. 2)).

Regarding potential barriers, just over half of the respondents feared that teaching students would lead to fewer patients being treated (53%). Only 28% of participants felt that the practice workflow was disrupted by students.

### 3.2. Analysis of factors influencing teaching motivation

While sociodemographic factors such as age, gender, years of medical practice, practice type, practice location, or previous teaching experience did not have a significant influence on teaching motivation (item: “How motivated are you to train medical students in your practice?”), statistically significant differences were identified regarding incentives and barriers in the univariate analysis (see table 2 (Tab. 2)). Physicians who feared that having students present would result in fewer patients being treated, or who felt that students disrupted the practice workflow, were significantly less motivated (p<0.0001 and p=0.00037). Motivation was significantly higher among respondents who believed that “both sides would benefit from knowledge exchange” or who viewed “training students as a responsibility toward society” (p=0.015 and p<0.0001).

**Figure 3:**
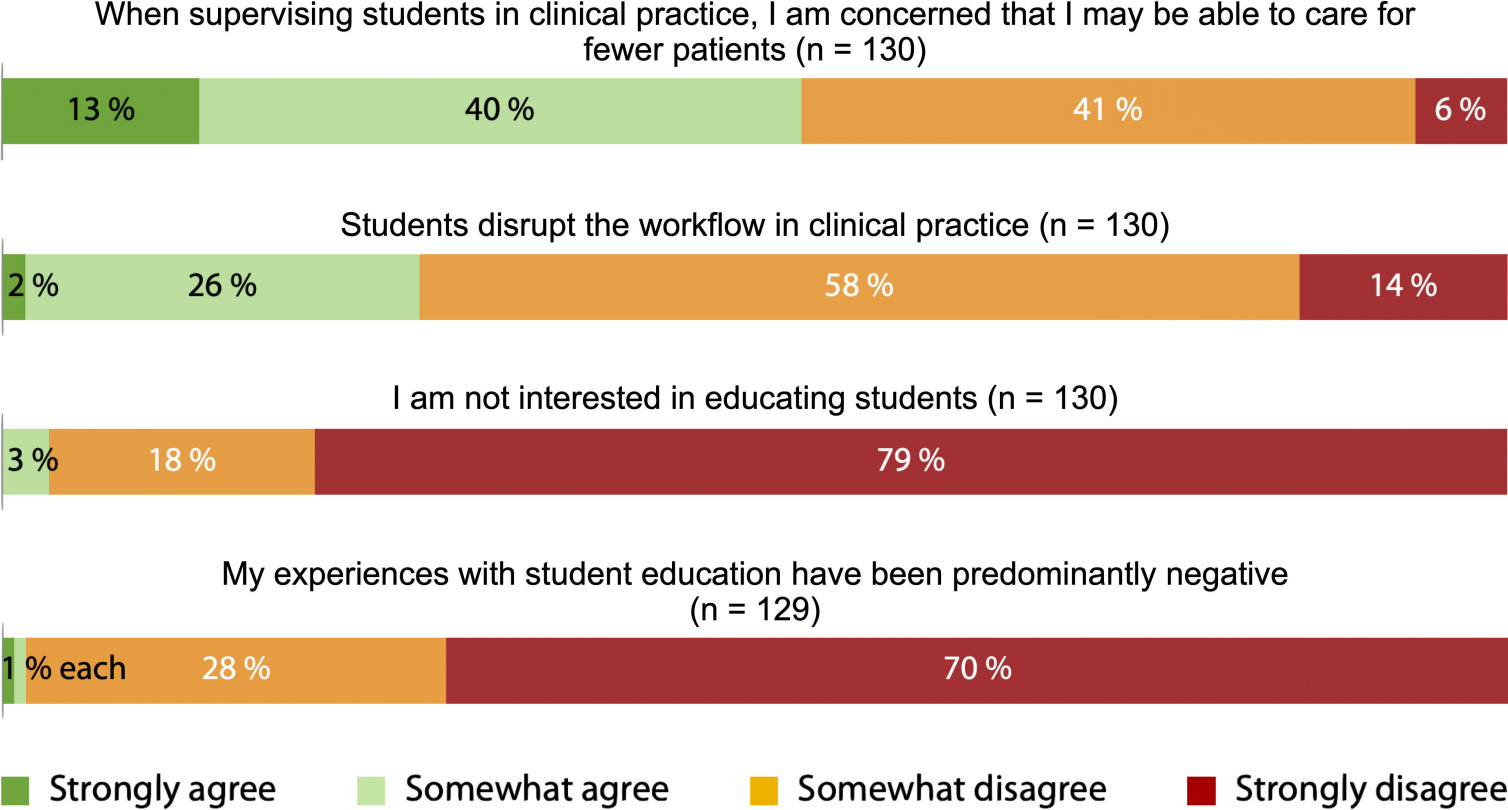
Agreement with barriers related to student teaching based on a 4-point Likert scale

Among the largest extrinsic motives leading to higher teaching motivation were the “positive impact of student teaching on patient satisfaction” (p=0.0067), the “enhancement of the practice with the designation “academic teaching practice”” (p=0.013), the “opportunity to form new contacts and networks” (p=0.016), and being “perceived as more competent by patients as a teacher” (p=0.014). Incentives such as “financial compensation”, the “increased chance of finding a successor for the practice”, and “participation in discounted or free educational courses” were not associated with significantly higher teaching motivation (see table 2 (Tab. 2) and figure 3 (Fig. 3)).

### 3.3. Analysis of subgroups: “experienced” vs. “inexperienced” in student teaching

The sample was dichotomized based on their teaching experience (whether they had previous experience with students in their practice; see table 1 (Tab. 1)). Seventy-five percent of the respondents had prior experience with students during their time in practice. The teaching motivation of the experienced group (n=99) did not differ significantly from that of the inexperienced group (n=33) (experienced: 7.6/10 vs. inexperienced: 7.2/10, p=0.14). Regardless of experience, the barriers to student teaching were assessed without significant differences.

However, differences emerged regarding the perceived benefits of being involved as a teaching practice. While approximately 64% of the experienced participants saw the opportunity to “find a successor for the practice” through teaching, only 39% of the inexperienced group agreed with this statement (p=0.024). Additionally, pediatricians with teaching experience in an outpatient practice more frequently viewed student teaching as a responsibility toward society during their time in practice (89.8% vs. 69.7%, p=0.0098). No significant differences were found between the two groups for any other incentives.

## 4. Discussion

The present study is the first to investigate the teaching motivation of outpatient pediatricians in the rural federal state of Lower Saxony using a comprehensive questionnaire survey. This motivation is predominantly driven by intrinsic factors, such as the desire to share knowledge and contribute to the education of future generations. The high level of intrinsic motivation aligns with previous findings on the teaching motivation of physicians, both in Germany and internationally. Studies by Latessa et al. [8] and Thomson et al. [9] confirm that intrinsic motives, such as the joy of knowledge exchange and a sense of social responsibility, are key drivers of physicians' willingness to teach. In the study by Deutsch et al. [13], over 80% of surveyed general practitioners stated that they were primarily motivated by idealistic values to train students in their practices. Comparable studies focusing on pediatricians are currently lacking.

Compared to the study by Daunert et al. [5], the level of intrinsic motivation among pediatricians in Lower Saxony appears to be similarly high as that of general practitioners in Thuringia. Both groups showed strong agreement on the importance of fostering young medical talent and promoting knowledge exchange. However, extrinsic motives seem to play a less significant role for pediatricians. As in Daunert et al. [5], opportunities for continuing education and the chance to engage in professional networks were also perceived as motivating. A potential explanation for these nuanced differences might lie in the distinct patient populations and daily workflows of pediatric versus general medical practices, which could require more tailored incentives.

The importance of early integration of students into primary care settings has been well documented internationally. In addition to fostering professional identity and increasing learning motivation, experiences in primary care also help to bridge the gap between theoretical knowledge and practical application [4]. Expert-based recommendations for relevant educational content have been published both internationally and nationally [2], [14].

### 4.1. Barriers and organizational challenges

The present survey illustrates that physicians with lower teaching motivation also report concerns about potential disruptions to practice operations and fear a decrease in the number of patients treated – even within the field of pediatrics. This finding is consistent with earlier studies in which physicians identified organizational and time-related challenges as key barriers to student education [15]. Baldor et al. similarly described concerns in general practice settings regarding potential limitations in patient care and increased organizational demands associated with teaching responsibilities [16].

When establishing teaching practices, it is therefore essential to evaluate practice workflows for the meaningful integration of students. Recommendations for the integration and structural requirements of teaching practices have been provided by Huenges et al. [2]. Collaborating with medical education experts appears beneficial, especially in light of rising patient numbers and increasing administrative demands, which continue to reduce the time available for the supervision of students [13], [17], [18].

Moreover, a future implementation of the CanMEDS competency framework – which defines key professional roles – would necessitate the integration of learning opportunities across both ambulatory and inpatient settings [19]. This poses particular challenges, as the inclusion of outpatient stakeholders in primarily university-based medical education also implicates institutional issues such as the allocation of faculty funding, legal capacity regulations, and personnel resources.

### 4.2. Strategies to promote teaching motivation

Fostering teaching motivation is essential to ensure high-quality, practice-oriented education for medical students [20]. In order to strengthen teaching motivation in pediatrics, various strategies can be considered. Studies indicate that structured didactic training and regular feedback sessions can enhance motivation to teach. Didactic training programs tailored to the needs of physicians in outpatient settings appear to be a promising approach [21]. Within the framework of a “pediatric teaching network”, joint training sessions focused on specific pediatric topics could contribute to the attractiveness of teaching activities.

Although not directly addressed in the current study, increased interdisciplinary collaboration may alleviate the burden on teaching physicians, as recommended by Ullian et al. [22] and Alberti and Atkinson [23], who emphasize the importance of professional networking and mutual support. Furthermore, targeted strategies that address both intrinsic and extrinsic motivational factors may foster greater engagement.

Intrinsic motivation is strongly supported by appreciation and recognition. The opportunity to act as a mentor and contribute to the next generation of physicians promotes a sense of meaningful involvement in medical education [8]. The implementation of regular feedback and recognition systems – through which teaching physicians receive insights into the developmental impact they have on their students – can further strengthen this intrinsic motivation. Additionally, networking events for teaching physicians can foster the exchange of experiences and best practices [21]. Invitations to university events such as a “teaching day” can promote public recognition. Providing didactic training opportunities can also enhance both teaching competence and confidence when working with students [9].

However, time and resource constraints – as well as access to training and supervision – can significantly influence the success and acceptance of these measures, particularly across different geographic regions and practice settings. In pediatrics, where specific challenges such as early detection and prevention are central, specialized professional development offerings can enhance intrinsic motivation by contributing to personal and professional growth. Access to scientific literature and educational opportunities – also advocated within the CanMEDS competency framework – can strengthen the sense of staying up to date with current knowledge [19].

A common barrier to teaching engagement is the concern that students might disrupt daily routines in outpatient practices. To address this concern, medical faculties could provide organizational support, for example through flexible teaching schedules or tools that facilitate the integration of students into clinical workflows [2], [15]. Close coordination with teaching physicians can help design processes that minimize disruption while maintaining high educational quality [24], [25].

Although financial remuneration was rated as a less significant motivator in the present survey, it may still play a supportive role when combined with other measures. Studies suggest that appropriate financial compensation does not necessarily diminish intrinsic motivation – particularly when it is perceived as a form of recognition for the additional workload and remains within a reasonable scope [26].

### 4.3. Limitations

The newly adapted questionnaire for pediatrics has not yet been validated. A major limitation of this study lies in the selection of participants. Due to the nature of the survey, a certain degree of response bias is likely, as those physicians who already had an interest in teaching may have been more inclined to participate. Individuals working simultaneously in hospital and outpatient settings (e.g., within structured postgraduate training networks) were not explicitly considered. Moreover, the design of the questions addressing motivation and barriers allows only limited differentiation, and open-ended responses were only partially possible.

Because no personal transaction number (TAN) was used, multiple submissions by the same individual cannot be completely ruled out as a potential bias. No formal training on the use of the questionnaire was provided to the target group. Additionally, situational factors such as individual workload or stress levels at the time of participation were not assessed and could have influenced the responses, thereby limiting the interpretability of the results.

For future research, qualitative interviews or focus groups could be used to capture a broader spectrum of responses and to explore individual motivations and concerns in greater depth - particularly with respect to differences between rural and urban settings. Furthermore, the participant sample was limited to the federal state of Lower Saxony, so regional effects may have influenced the results. An exact response rate cannot be determined, as some contacted practices employed multiple physicians, and it remains unclear whether more than one physician from the same practice took part in the survey.

## 5. Conclusions

This study is the first to demonstrate that the teaching motivation among pediatricians is generally high; however, organizational and structural challenges need to be addressed. Previous studies emphasize that targeted support, clear recommendations for action, and organizational measures – such as flexible teaching models or financial incentives – are essential to sustaining long-term motivation [27]. Promoting intrinsic motivation, alongside organizational support and material recognition, can help strengthen the willingness to teach in outpatient pediatrics over the long term. Close cooperation between universities and teaching physicians is indispensable for building a sustainable teaching network.

Future research should examine the resources required to support teaching, assess how the growing shortage of healthcare professionals affects teaching conditions, and explore how specific topics such as patient safety or rare diseases can be addressed in a context-sensitive manner.

## Notes

### Funding

This work was supported by the Stiftung Innovation in der Hochschullehre as part of the “Freiraum 2023” program (FR-459/2023 Pedagotchi).

### Authors’ ORCIDs

- Alexandros Rahn: [0009-0003-9227-7938]
- Thomas Müller: [0009-0009-0762-2420]
- Urs Mücke: [0000-0002-0240-248X]

## Acknowledgements

The authors would like to express their sincere gratitude to the German Association of Paediatricians (Berufsverband der Kinder- und Jugendärzt*innen e.V., BVKJ), the German Society for Outpatient General Paediatrics (Deutsche Gesellschaft für Ambulante Allgemeine Pädiatrie, DGAAP e.V.), and the Association of Statutory Health Insurance Physicians of Lower Saxony (Kassenärztliche Vereinigung Niedersachsen, KVN) for their valuable support in conducting this survey.

Special thanks go to all colleagues who contributed to the improvement of medical education by participating in the study.

## Competing interests

The authors declare that they have no competing interests. 

## Figures and Tables

**Table 1:**
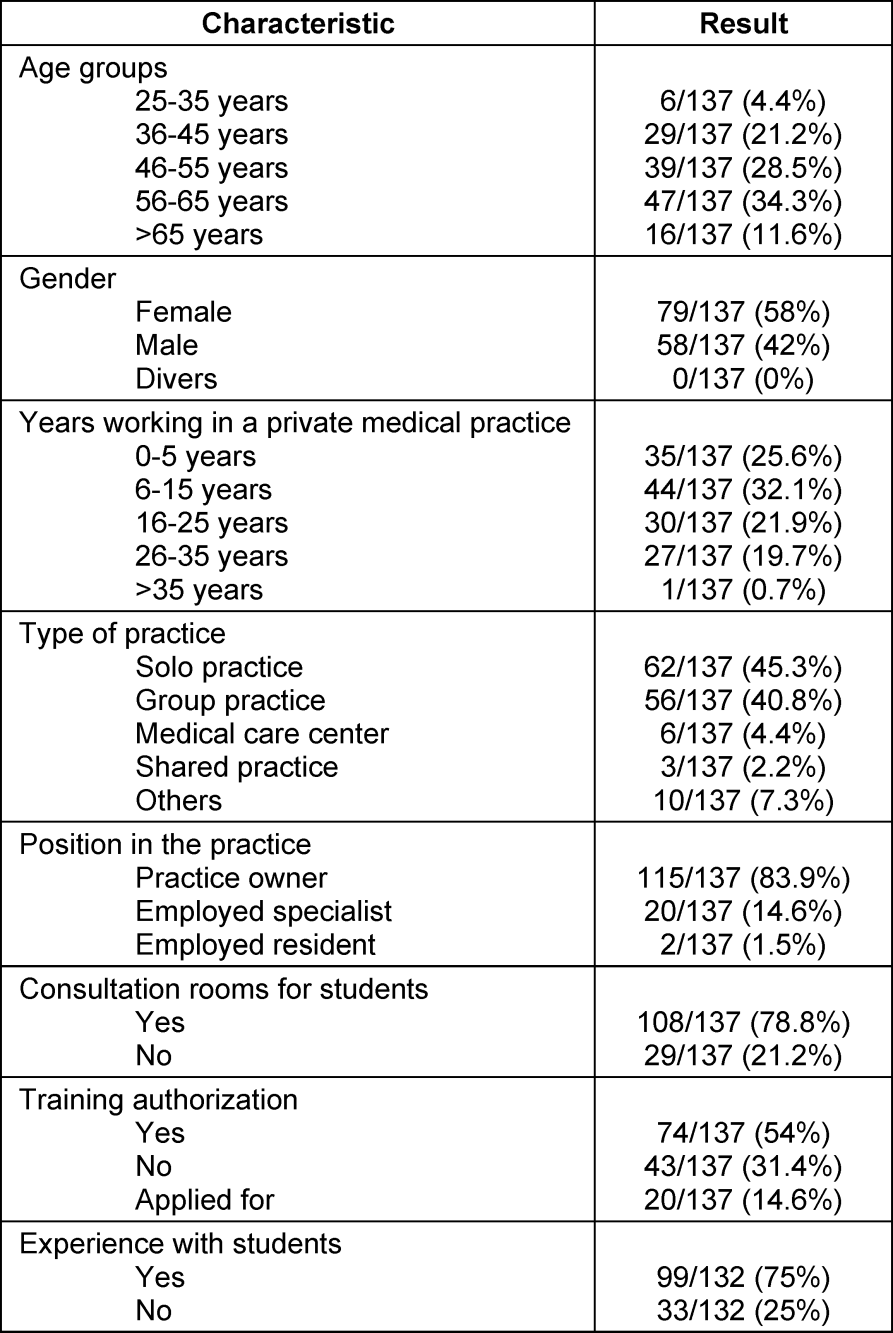
Sociodemographic data of the 137 evaluable participants in the survey (selection)

**Table 2:**
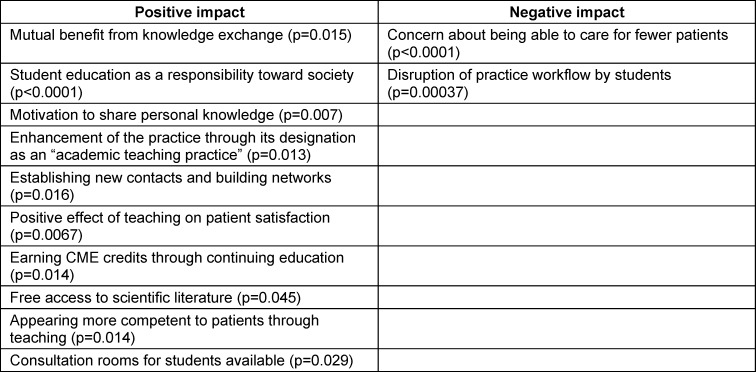
Factors influencing motivation to teach (selection of significant items)
